# High‐Frequency Changes in Shopping Behaviours, Promotions and the Measurement of Inflation: Evidence from the Great Lockdown*

**DOI:** 10.1111/1475-5890.12241

**Published:** 2020-11-30

**Authors:** Xavier Jaravel, Martin O'Connell

**Affiliations:** ^1^ London School of Economics; ^2^ Institute for Fiscal Studies

**Keywords:** inflation, Great Lockdown

## Abstract

We use real‐time scanner data in Great Britain during the COVID‐19 pandemic to investigate the drivers of the inflationary spike at the beginning of lockdown and to quantify the impact of high‐frequency changes in shopping behaviours and promotions on inflation measurement. Although changes in product‐level expenditure shares were unusually high during lockdown, we find that the induced bias in price indices that do not account for expenditure switching is not larger than in prior years. We also document substantial consumer switching towards online shopping and across retailers, but show this was not a key driver of the inflationary spike. In contrast, a reduction in price and quantity promotions was key to driving higher inflation, and lower use of promotions by low‐income consumers explains why they experienced moderately lower inflation. Overall, changes in shopping behaviours played only a minor role in driving higher inflation during lockdown; higher prices were the main cause, in particular through a reduced frequency of promotions.

## I. Introduction

The COVID‐19 pandemic led many countries to implement lockdowns, resulting in a worldwide economic crisis. Accurate measurement of inflation in real time is crucial to monitor inflation risks and adjust policies accordingly. As the Great Lockdown entails a combination of substantial shocks to both demand and supply, the crisis may lead to deflation, disinflation or higher inflation. Whilst falling aggregate demand may lead to deflationary pressures, inflationary pressures may arise from increases in production costs due to interrupted supply chains and to the impact of social distancing restrictions on labour supply. It is therefore essential to accurately measure how prices have actually evolved during the crisis and to isolate the main factors driving price changes.^1^ For example, Jaravel and O'Connell (2020) use comprehensive scanner data from Great Britain to measure inflation during the Great Lockdown in real time and document a significant and widespread spike in inflation.

An emerging literature^2^ analyses biases in inflation measurement that may arise over this crisis due to the methodology and data used to construct standard inflation statistics. As noted by Cavallo (2020), Diewert and Fox (2020) and Seiler (2020), the COVID‐19 pandemic has led to large and sudden changes in consumers’ expenditure patterns. These substitution effects are not accounted for by standard inflation measures, because most national statistical offices update the expenditure weights used in their official consumer price index (CPI) annually, typically with lagged expenditure data. Changes in expenditure shares across products, across retailers and across online and offline outlets may all lead to biased inflation measures.

Concerns over biases arising from expenditure switching have a long history in price index theory, going back to Gerschenkron (1947). Failure to account for expenditure switching typically leads to upward bias in price indices, but little is known regarding whether the bias differs during periods when changes in expenditure patterns are unusually large, such as during the COVID‐19 pandemic. In normal times, consumers tend to substitute toward items that become relatively cheaper over time, meaning that standard indices that do not account for expenditure switching tend to overstate inflation. The bias could be very different during the COVID‐19 pandemic, because of the unprecedented nature of the crisis. For example, due to social distancing and higher search costs, consumers may have altered their shopping behaviours during lockdown in such a way that they now obtain the same products from more expensive outlets.

In this paper, we use real‐time barcode‐level data covering fast‐moving consumer goods in Great Britain to assess to what extent high‐frequency changes in shopping behaviours influence and create biases in inflation measurement. We thus extend the analysis of Jaravel and O'Connell (2020), who use these data to document a large spike in inflation in the fast‐moving consumer goods segment at the beginning of lockdown. In the first month of lockdown, month‐to‐month inflation was 2.4 per cent. This sharp inflationary spike is unprecedented in prior years and represents more inflation than is typical in a year. Here, we analyse to what extent the inflation spike is tied to changes in shopping behaviour and promotions.

We organise the analysis in three parts. In the first part (Section III), we compare inflation measures obtained with alternative price indices, which allow for expenditure switching to different degrees. Cavallo (2020) and Seiler (2020) document that changes in expenditure patterns across broad sectors during the pandemic led to an increase in inflation experienced by consumers. Their results are driven by the relative increase in consumption of food and non‐alcoholic beverages, which are more inflationary than other spending categories. While these papers focus on expenditure switching patterns across broad sectors, we study the same substitution bias channel within the fast‐moving consumer goods sector, using a real‐time scanner data set where prices and quantities are precisely measured.^3^

Using a dissimilarity index based on barcode‐level expenditure, we confirm that expenditure patterns (within fast‐moving consumer goods) changed more substantially than usual during the pandemic. However, perhaps surprisingly, we find that the degree of substitution bias is not larger than in prior years. Using a Laspeyres index relative to a superlative (for example, Fisher) index leads to higher inflation but the bias is very similar in magnitude in 2018, 2019 and 2020.

In the second part of the analysis (Section IV), we analyse two specific changes in shopping behaviour: changes in shopping format (in particular, online versus offline purchases) and changes in expenditure shares across retailers. The disruptions caused by lockdown may have led consumers to alter their shopping behaviours in ways that mean they now obtain the same products from more expensive outlets. We show that lockdown coincided with a significant increase in the use of online shopping and a more modest increase in shopping in small store formats. However, we find that these changes in shopping behaviour occurred mostly after the inflation spike and cannot account for it, in particular because online and offline prices for identical items are similar. We also show that expenditure switching across retailers (for the same barcodes) played a small role in driving aggregate inflation; although there was some substitution towards more expensive convenience stores, the extent of the switching and the price differential compared with other stores are too small to contribute to a large increase in measured inflation.

These new facts can help explain why the changes in expenditure patterns observed during lockdown – in particular, the large rise in online purchases – do not affect inflation measurement substantially in practice. In contrast, a fall in the frequency of promotions accounts for a large share of the inflation spike. We refine the findings of Jaravel and O'Connell (2020) on promotions by presenting additional evidence on the roles of price promotions and quantity discounts. We find that changes in both promotion types are substantial, although there are marked differences across retailers. In the first month of lockdown, compared with the same period in the preceding year, an additional 5.5 percentage points of consumer spending entailed transactions in which there was no promotion. Around two‐thirds of this rise is accounted for by a reduction in the share of expenditure entailing a price promotion; the remaining third is due to a reduction in quantity promotions. The reduction in promotional activity is most pronounced in large full‐line national supermarket chains and premium supermarket chains.^4^ These findings indicate that it is crucial for statistical agencies to measure promotions accurately, especially during major economic crises.^5^ Within‐sector substitution patterns appear to be an important but more generic cause of substitution bias in inflation measurement, whose magnitude has remained unchanged during lockdown.

In the third part of the analysis (Section V), we examine whether changes in promotions and shopping behaviour may impact inflation measurement differentially across household groups. We document the extent of heterogeneity across households based on a measure of their permanent income (their equivalised total spending). We find that the patterns of substitution across retailers and shopping formats are broadly similar across income groups, while the fall in promotions affected high‐income households more. During the first month of lockdown, households in the bottom quartile of the spending distribution had an inflation rate that was 20 basis points below the average. We show that this can be accounted for by a more modest reduction in the use of price and quantity promotions for this group. These households tend to use sales less than higher‐spending households; therefore their baskets are less exposed to higher inflation driven by a reduction in promotional activity. These findings underscore the importance of measuring promotions accurately.

Overall, the results indicate that, in practice, the sudden changes in expenditure patterns during the pandemic did not lead to unusually large biases in inflation measurement. Changes in shopping behaviours played only a minor role in driving inflation; higher prices were the main cause, in particular through a reduced frequency of promotions. Our findings raise potential concerns about business dynamism and competition going forward. We find that the market shares of the largest retailers have increased during the crisis. Furthermore, the online marketplace is more concentrated than brick‐and‐mortar retailers, and its increased share of total transactions will likely be sustained. Real‐time scanner data can be used to monitor changes in market concentration going forward.

## II. Method

### 1. Data

We use household‐level scanner data that are collected by the market research firm Kantar FMCG Purchase Panel. The data cover purchases of fast‐moving consumer goods brought into the home by a sample of households living in Great Britain (i.e. the UK excluding Northern Ireland). Fast‐moving consumer goods include food and drinks (including alcohol), as well as cleaning products, pet foods and toiletries. At any point in time, the data set contains purchase records of around 30,000 households. Participating households are typically in the data for many months. Each household records all UPCs (universal product codes, or barcodes) that they purchase using a hand‐held scanner, and they send their receipts (either electronically or by post) to Kantar. For each transaction, we observe quantity, expenditure, price paid, UPC characteristics and store characteristics.^6^

Our data set runs until 17 May 2020. In the UK, lockdown started on 23 March 2020. This entailed the closure of non‐essential stores and eat‐in restaurants and bars. Stores specialising in fast‐moving consumer goods, such as supermarkets, convenience stores and off‐licences, were permitted to remain open. We focus on the period from the beginning of the year to 17 May. Over this period in 2020, we observe 13.4 million transactions and 102,000 distinct UPCs.^7^ We measure month‐to‐month inflation, defining months as running from the 18^th^ of one month to the 17^th^ of the following month. We focus on the five months running from 18 December to 17 May.

A goal of our analysis is to unpack to what extent changes in shopping behaviour contributed to the inflationary spike documented in Jaravel and O'Connell (2020) in the first month of lockdown. We use information on the store a transaction took place in to construct a classification of transactions into ‘shopping format’ and ‘retailer type’ (see the first two columns of Table 1). Our shopping format classification uses store information to separate transactions into those that took place in large stores, in compact stores, via internet shopping and in stores that specialise in non‐food produce.^8^ The retailer type classification splits transactions based on the retailer in which they took place (whether this be in‐store or online). ‘Big four’ refers to transactions that took place with one of the dominant full‐line UK supermarkets (Asda, Morrisons, Sainsbury's and Tesco); ‘discounters’ refers to transactions that took place in national supermarket chains that focus on providing products at low prices (for example, Aldi, Iceland and Lidl); ‘premium’ refers to national retailers that focus on high‐end products (for example, Marks and Spencer, Ocado and Waitrose); ‘convenience’ refers to national and local retailers that sell food and have a small convenience store format; and ‘non‐food’ refers to retailers that specialise in non‐food produce.

**TABLE 1:** Shopping behaviours and promotion status classification

*Shopping format*		*Retailer type*		*Promotion status*	
Large stores	(78.3%)	Big four	(63.2%)	No promotion	(67.4%)
Compact stores	(5.7%)	Discounters	(22.2%)	Price promotion	(22.5%)
Internet	(11.3%)	Premium	(5.3%)	Quantity promotion	(10.1%)
Non‐food stores	(4.7%)	Convenience	(4.5%)		
		Non‐food	(4.8%)		

*Note*: Share of expenditure in 2019 is given in parentheses.

We also explore the role played by changes in the share of transactions on promotion in driving inflation. We split transactions into those entailing no promotion, those entailing a price promotion (for example, £1 off) and those entailing a quantity promotion (for example, 2 for £2, 3 for 2, or 20% extra) – see the third column of Table 1. We thus extend the analysis of Jaravel and O'Connell (2020), who showed the importance of changes in promotions but did not distinguish between quantity and price promotions.

### 2. Price index

Price indices entail weighting product price changes between two periods using expenditure weights. In our analysis, we use an index that focuses on continuing products. We also make the distinction between a chained and a fixed base index, and use a superlative index.

Most standard price indices (including those used by national statistical offices) focus on price changes of *continuing products*. This means they compare the price of products continuously available over some particular time horizon, not accounting for the impact of product entry or exit on the cost of living.^9^

An index that is *chained* uses expenditure weights that are updated each period (in our case in each month). This contrasts with a *fixed base* index (commonly used by national statistical offices), which holds expenditure weights fixed at some reference value. The advantage of chained indices is that they capture changes in households’ expenditure patterns, providing a better approximation to a true cost‐of‐living index. This may be particularly important during lockdown, where there are likely to be substantial changes in consumer spending.^10^

We use a *superlative* index. This means the index uses some combination of past and current/final‐period expenditures in the weights, and provides a second‐order approximation to a true cost‐of‐living index. In particular, we use the Fisher index, which is a geometric mean of the (non‐superlative) Laspeyres and Paasche indices, which use expenditure weights in a base and in the current/final period respectively.^11^

We first define the chained version of our price index. Let *i* denote all products present in two successive months, *t* and *t*+1. Denote by *p_i_*_,_ *_t_* the average price of product *i* in period *t* 12 and denote by *q_i_*_,_ *_t_* the total quantity of product *i* at time *t*. The chained Fisher price index is defined as

$$1+\pi_{t,t+1}^{Fisher}\equiv \sqrt{\left(1+\pi_{t,t+1}^{Laspeyres}\right)\left(1+\pi_{t,t+1}^{Paasche}\right)},$$

where $1+\pi_{t,t+1}^{Laspeyres}\equiv \frac{\sum_{i}q_{i,t}\cdot p_{i,t+1}}{\sum_{i}q_{i,t}\cdot p_{i,t}}$ and $1+\pi_{t,t+1}^{Paasche}\equiv \frac{\sum_{i}q_{i,t+1}\cdot p_{i,t+1}}{\sum_{i}q_{i,t+1}\cdot p_{i,t}}$; $\pi_{t,t+1}^{Fisher}$ denotes the rate of inflation between periods *t* and *t*+1. The fixed base index is defined similarly, but with two modifications: (i) the product set, *i*, is those products present in all months *t* = {1,…,*T*}; and (ii) the quantity weights used are those in the first and final periods of data (i.e. the Laspeyres index is computed using *q_i_*_,1_ and the Paasche index is computed using *q_i_*_,_ *_T_*).

An important advantage of using scanner data to measure inflation is that they allow us to define the products at a very disaggregate level. For most of the analysis, we define products as UPCs. In robustness checks, we use alternative product definitions: UPC–store‐format and UPC–retailer. This helps us isolate the impact of changes in shopping behaviour on measured inflation.

## III. Substitution bias during the lockdown

Cavallo (2020) and Diewert and Fox (2020) point out that the COVID‐19 pandemic has led to large and sudden changes in consumers’ expenditure patterns. These substitution effects are not accounted for by standard inflation measures, because most national statistical offices update their CPI expenditure weights once a year, typically with lagged expenditure data.

In normal times, failure to account for expenditure switching typically leads to upward bias in price indices. As consumers typically substitute toward items that become relatively cheaper over time, failing to capture expenditure switching tends to overstate inflation. However, the COVID‐19 pandemic features unusual and atypically large shifts in expenditure patterns, and it is unclear whether these will exacerbate substitution bias. For instance, social distancing requirements and ‘stay at home’ orders led consumers to greatly alter their shopping behaviours during lockdown. Because of higher search costs compared with normal times, consumers may have switched towards sellers that charge higher prices for identical items. This raises the possibility that expenditure switching could lead to a downward bias in standard price indices, which keep expenditure weights at their pre‐crisis levels.

**FIGURE 1:**
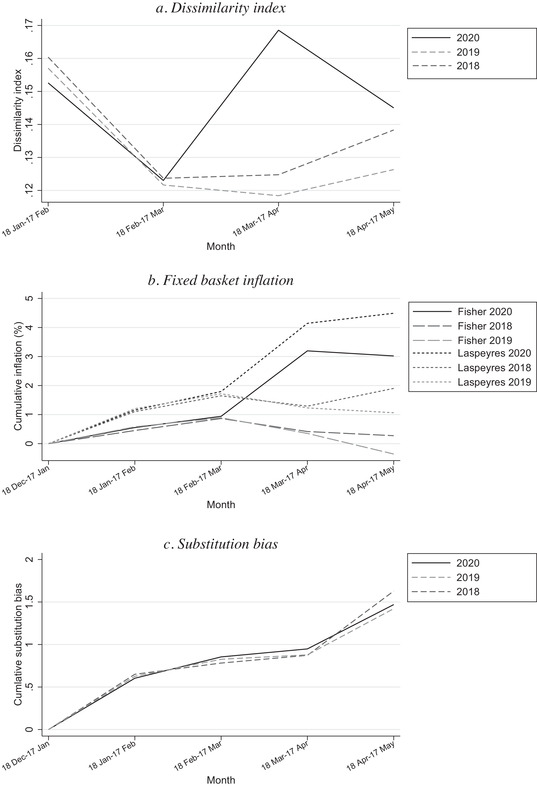
Expenditure switching across products and substitution bias *Note*: Panel a shows a month‐to‐month index of dissimilarity for UPCs continuously available across the first five months of the year. Panel b shows cumulative monthly inflation measured with the fixed base Laspeyres and Fisher indices. Panel c shows the difference in cumulative inflation measured based on the fixed base Laspeyres and Fisher indices.

In panel a of Figure 1, we use a dissimilarity index based on UPC‐level expenditure to examine whether expenditure patterns indeed changed more substantially than usual during the pandemic, within the set of fast‐moving consumer goods. The dissimilarity index is computed from one month to the next, for barcodes available across all five months, and captures the magnitude of changes in expenditure shares across UPCs over time.^13^ The figure shows that in 2020 12.5 per cent of spending needs to be reallocated for the distribution of barcode expenditure shares from 18 February to 17 March to match the distribution observed from 18 January to 17 February. The dissimilarity index increases to 17 per cent during the first month of lockdown, and falls back to 14.5 per cent the following month. In 2018 and 2019, in contrast, the dissimilarity indices were stable from the month 18 February to 17 March onward, at around 12–13 per cent. These patterns indicate that there was a substantial switch in expenditures at the onset of lockdown, which is the time when inflation spiked. The dissimilarity index is about 40 per cent larger than in prior years.^14^

In panel b, we compare fixed base Laspeyres and Fisher indices across all five months in 2018, 2019 and 2020. Compared with prior years, we find that inflation spikes at the beginning of lockdown for both the Laspeyres and Fisher indices (as documented in Jaravel and O'Connell (2020)). In panel c, we compute the cumulative bias in measured inflation when using the fixed base Laspeyres index (which does not allow for reallocation of expenditure) rather than the fixed base Fisher (which is a superlative index accounting for expenditure switching). The bias is strikingly similar in all three years. After five months, the Laspeyres index overstates inflation by about 1.5 percentage points in all three years. This is a large bias, arising from the fact that consumers reallocate their expenditure toward items that become relatively cheaper over time. Perhaps surprisingly, the bias is not larger during lockdown, despite the larger reallocation of expenditure shown in panel a. By 17 May 2020 relative to 18 December 2019, cumulative inflation is 4.5 per cent when measured with the Laspeyres index and 3 per cent based on the Fisher index, compared with 1 per cent with the Laspeyres index and –0.4 per cent with the Fisher index for 18 December 2018 to 17 May 2019, and 1.9 per cent with the Laspeyres index and 0.3 per cent with the Fisher index for 18 December 2017 to 17 May 2018.

These results show that the substitution bias is large but was almost identical in 2018, 2019 and 2020, and they suggest that changes in shopping behaviour, as reflected in changing UPC expenditure share, during the pandemic do not account for the inflationary spike during lockdown.

## IV. Mechanisms

In this section, we analyse specific mechanisms that help explain what did and did not drive the inflationary spike at the beginning of lockdown. We first analyse changes in shopping format (in particular, online versus offline purchases), then turn to changes in expenditure shares across retailers. We find that changes in these shopping behaviours were substantial, but differences in prices for identical goods across these retailers and shopping formats are too small to contribute to a large increase in aggregate inflation. Finally, we show that the reduced frequency of price and quantity promotions was the main cause of higher inflation.

### 1. Expenditure switching across shopping formats

Consumers changed how and where they shop during lockdown. Given that lockdown entailed strict social distancing rules, which led to widespread queuing outside stores, and that people were encouraged to work from home and to shop locally, it is likely that consumers may have switched toward online shopping or smaller local stores.

**FIGURE 2:**
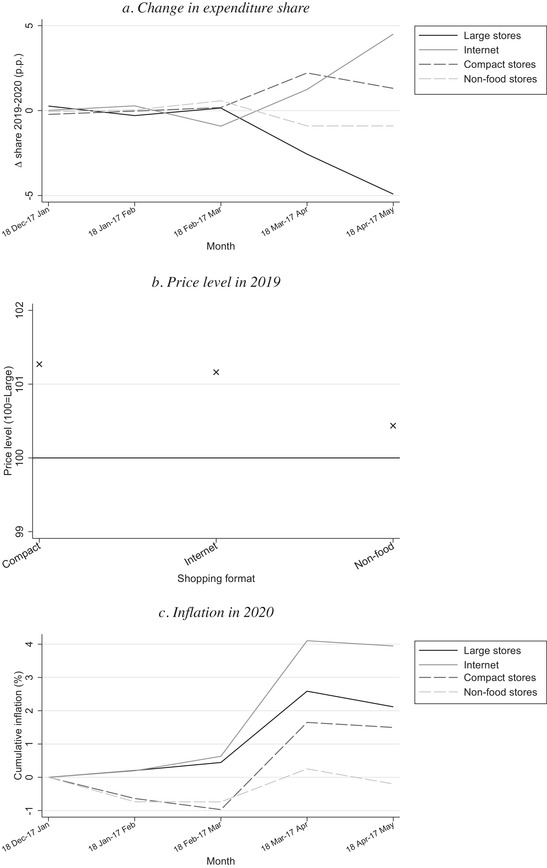
Shares and prices, by shopping format *Note*: Panel a shows change in expenditure share in percentage points in 2020 relative to 2019. Panel b shows difference in price level in 2019 in shopping formats relative to ‘large stores’. Panel c shows cumulative monthly inflation measured with a chained Fisher index.

In Figure 2, we investigate the role of changes in shopping format. We compare the change in expenditure shares, price levels in 2019 and inflation in 2020 across four types of shopping format: large stores, compact stores, online purchases and non‐food stores. Panel a shows the change, in percentage points, in the share of expenditure allocated to each shopping format in the first five months of 2020 relative to the corresponding month in 2019. There is a large increase in online purchases; in the month 18 April to 17 May, online purchases are 5 percentage points larger (relative to a baseline of around 11 per cent). There is also an increase in the share of expenditure in compact stores. Large stores exhibit the largest fall in share, and there is a modest fall for non‐food stores.

Panel b shows how the price level differs across shopping formats in 2019. We measure differences in price levels relative to large stores, the largest category. When comparing the price level with the baseline category, we compare the expenditure‐weighted average price for the set of identical UPCs common to both shopping format types. For example, consider the comparison of the price level in compact store and large store shopping formats. Let *i* denote the set of UPCs we observe purchased in both formats in 2019. The price level of compact stores relative to large stores is given by $\sum_{i}p_{i}^{C}s_{i}/\sum_{i}p_{i}^{L}s_{i}$, where $p_{i}^{C}$ and $p_{i}^{L}$ denote the average unit price in 2019 of UPC *i* in transactions classified as shopping format compact stores and large stores respectively, and *s_i_* denotes the share of total spending in these shopping formats allocated to UPC *i*.

Panel b shows that the shopping formats that gained market shares – online purchases and compact stores – are about 1 per cent more expensive on average for identical barcodes compared with large stores. The finding that online and offline prices are very similar is consistent with Cavallo (2017). These modest differences in price levels mean that, even though the changes in expenditure shares shown in panel a are considerable, switching across shopping formats makes little contribution to overall inflation.^15^

Finally, panel c plots inflation in 2020 within each shopping format. The inflation spike at the onset of lockdown is about 3.5 percentage points for online purchases, 2 percentage points for large stores, 2.5 percentage points for compact stores and 1 percentage point for non‐food stores. Combined with panel a, these patterns indicate that, interestingly, consumers substituted toward shopping formats where inflation was higher (online purchases and compact stores), not lower. This fact can be reconciled with consumer optimisation by taking into account changes in the shopping experience across shopping format. For example, perceived health risks may be higher for larger stores than for compact stores, while online purchases are particularly convenient during lockdown; this can explain why consumers substitute toward these outlets despite slightly higher inflation. These patterns show that changes in shopping behaviour during lockdown depend on changes in the shopping experience, not only on prices. Despite these noteworthy differences, the path of inflation is broadly similar across all shopping formats.

Although switching across shopping formats is substantial, the most pronounced substitution occurred after the significant inflation in the first month of lockdown (panel a), and relatively similar price levels (panel b) and inflation paths (panel c) across shopping formats mean this did not contribute much towards the overall inflationary spike.

### 2. Expenditure switching across retailer types

**FIGURE 3:**
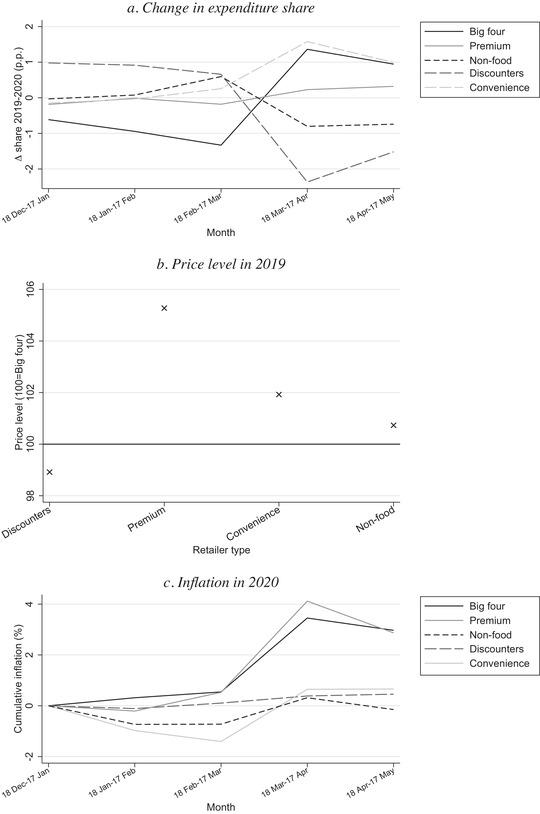
Shares and prices, by retailer type *Note*: Panel a shows change in expenditure share in percentage points in 2020 relative to 2019. Panel b shows difference in price level in 2019 in retailer types relative to ‘big four’. Panel c shows cumulative monthly inflation measured with a chained Fisher index.

We now turn to the role of expenditure switching across retailers, reporting the results in Figure 3. Panel a shows there were considerable shifts in the market shares of different retailer types and, in contrast to switching across shopping formats, the largest changes occurred in the month 18 March to 17 April, which coincides with the spike in inflation. The big four retailers and the set of convenience stores exhibit an increase in their market share in excess of 1 percentage point relative to the same month in 2019, premium retailers also exhibit a (smaller) increase, while discounters exhibit a reduction of 2 percentage points.

Panel b shows how price levels in 2019 differ across retailer types. We draw the comparisons relative to the big four retailers, using the set of UPCs common to both retailer types. Premium retailers are the most expensive, with prices that are about 5 per cent higher for identical UPCs. Convenience stores are also more expensive than the big four, with a price level about 2 per cent higher. Despite their reputation for low prices, the set of discounter retailers have only a marginally lower price level than the big four. The reason is that the comparison of price levels is based only on UPCs available in both sets of retailer types (which represent only 15 per cent of spending in discounters); the reputation of these retailers for low prices is in large part based on their store brands, which are UPCs unique to the discounter retailer type.

Panel c shows that the paths of inflation in retailers belonging to the big four and in premium retailers are very similar. Convenience stores also exhibit significant inflation in the first month of lockdown, while other retailer types do not exhibit significant inflation. Combined with panel a, these patterns indicate that consumers substituted toward some retailer types that became more expensive – the big four and premium retailers. This may be due to changes in the shopping experience during lockdown. However, the switching across retailer types and the differences in cross‐retailer‐type prices are too small to play a major role in driving the inflationary spike.^16^

The combination of significant price increases and changes in retailer market shares points towards a potential concern that competition in the market is being eroded. The restrictions of lockdown mean that consumers are more likely to shop locally, potentially benefiting large retailers with extensive networks of stores, while the switch towards online purchases is also likely to benefit larger supermarkets that already have established online delivery operations. Such concerns are heightened by a relaxation of competition restrictions over the COVID‐19 crisis.^17^ Nevertheless, as the lockdown restrictions are eased, shopping patterns may return to normal. In addition, the price inflation over lockdown does not necessarily reflect a lessening of competition and may, for instance, be driven by higher costs. It is important, however, that policymakers monitor conditions in the market closely going forward.

### 3. The role of quantity and price promotions

**FIGURE 4:**
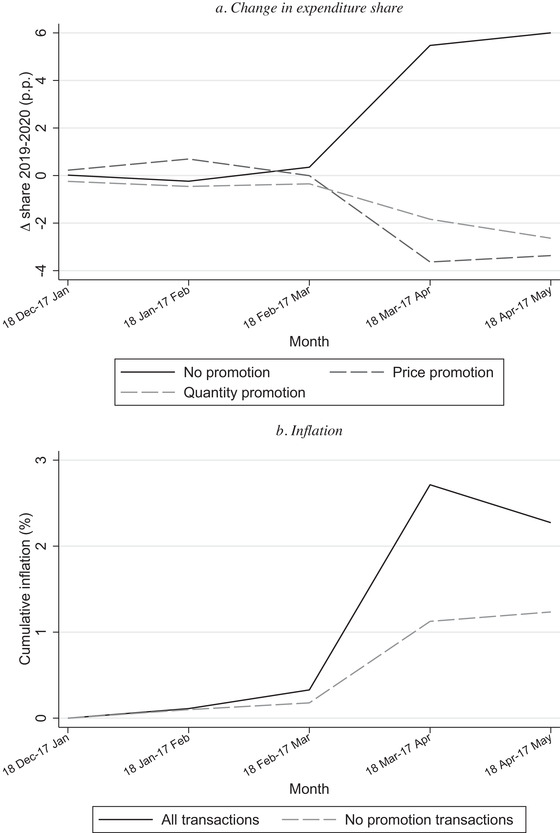
Inflation and promotions *Note*: Panel a shows change in expenditure share in percentage points in 2020 relative to 2019. Panel b shows cumulative monthly inflation measured with a chained Fisher index, based on all transactions and only non‐promotion transactions.

**FIGURE 5:**
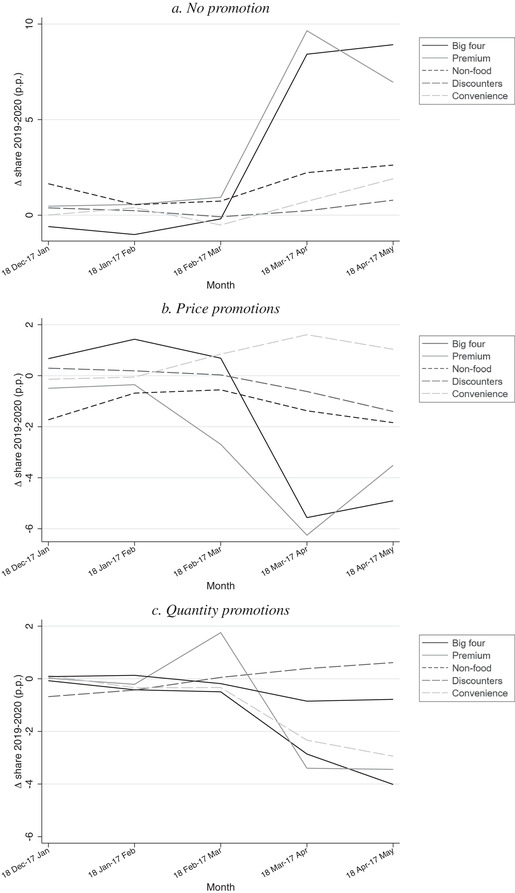
Promotions, by retailer type *Note*: Each panel shows change in expenditure share in percentage points in 2020 relative to 2019.

Jaravel and O'Connell (2020) document that at the beginning of lockdown there was a reduction in the share of transactions that were on promotion and they suggest this was an important contributing factor to the inflationary spike at the beginning of lockdown. In Figures 4 and 5, we explore further the role played by changes in promotions.

Panel a of Figure 4 shows the change in the shares of total expenditure that entailed no promotion, a price promotion and a quantity promotion, in the first five months of 2020 relative to the corresponding month in 2019. It shows that in the first month of lockdown, 18 March to 17 April, there was an additional 5.5 percentage points of total expenditure that entailed no promotion compared with the same period in the previous year. Around two‐thirds of this rise is accounted for by a reduction in the share of expenditure entailing a price promotion; the remaining third is due to a reduction in quantity promotions. These patterns indicate that it is important for statistical agencies to track both types of promotions.

In panel b, we show that inflation computed based only on transactions that entail no promotion was significantly lower than inflation computed using all transactions (including promotional ones); the inflation spike in the month 18 March to 17 April is 0.94 percentage points when computed based on non‐promotion transactions (compared with 2.4 percentage points based on all transactions). This highlights the central role changes in promotions played in driving the spike in inflation.

Figure 5 explores how differences in promotional activity vary across retailer types. Panel a focuses on the share of expenditure within each retailer type that entailed no promotion, and shows the change for each of the first five months of 2020 relative to the corresponding month in 2019. Panels b and c show the same information for price promotions and quantity promotions. The figure shows that the decline in promotional activity was mainly driven by the big four and premium retailers; in the first month of lockdown, both exhibit a reduction in price promotions of over 5 percentage points and a reduction in quantity promotions of over 3 percentage points. Convenience and non‐food stores exhibit smaller reductions in promotional activity, with the fall in convenience stores being driven by fewer quantity discounts. The discounter retailers exhibit little change in promotional activity, reflecting that these retailers tend to focus on an ‘every day low pricing strategy’. In Figure A.2 in the online appendix, we show how promotional activity changed across 13 broad product types; there was a reduction in promotional activity for each of these.

In sum, the facts presented in this section can help explain why the changes in expenditure patterns observed during lockdown – in particular, the large rise in online purchases – do not affect inflation measurement substantially in practice. In contrast, the fall in the frequency of promotions can account for a large share of the inflation spike. Next, we turn to heterogeneity across income groups.

## V. Heterogeneity by income group

In this section, we document heterogeneity in inflation across households based on a measure of their permanent income and illustrate the role of differences in promotions across the permanent income distribution in explaining inflation heterogeneity.^18^

The preceding analysis documents inflation experienced by the representative household, meaning that the product weights reflect expenditure shares computed across all households. However, because different types of households are likely to purchase different products, their experience of inflation may vary.^19^

We measure households’ permanent income as follows. For each household, we compute their total expenditure on fast‐moving consumer goods in 2019 and equivalise this using the standard OECD scale,^20^ and we split households into quartiles of the equivalised expenditure distribution. For brevity, we refer to these quartiles as ‘spending quartiles’. For each quartile, we compute inflation over the first five months of 2020. Inflation may differ across quartiles both because of differences in the index weights (spending patterns of rich and poor differ) and because of differences in prices paid.

**FIGURE 6:**
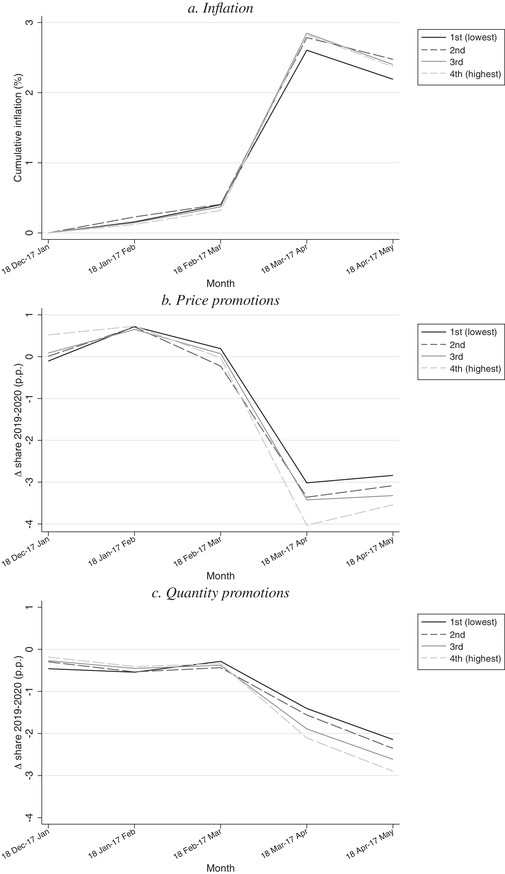
Inflation and promotions, by spending quartile *Note*: Panel a shows cumulative monthly inflation measured with a chained Fisher index. Panels b and c show change in share of expenditure on price and quantity promotions in percentage points in 2020 relative to 2019.

Panel a of Figure 6 plots inflation for each spending quartile. It shows that the inflationary spike in the first month of lockdown was experienced by all quartiles. However, there are some differences in the size of the spike across quartiles; for the lowest spending quartile inflation in the month 18 March to 17 April is 2.2 per cent, whereas for the higher spending quartiles it is between 2.4 and 2.5 per cent. Therefore, the poorest households experienced less inflation at the beginning of lockdown, though differences in inflation across the quartiles are small.^21^

Panel b shows, for each quartile, the change in the share of total expenditure on transactions that entail a price promotion for each of the first five months of 2020 relative to the corresponding month in 2019. Panel c shows changes for quantity promotions. In each case, there is a monotonic ordering across spending quartiles; the decline in their share of expenditure allocated to price and quantity promotions is smaller for lower spending quartiles.

Figure 6 suggests that an important reason why the lowest spending quartile experienced somewhat lower inflation than other households is that they exhibit smaller reductions in how much of their spending went on promoted items. The lowest‐income households, on average, spend less on goods on promotion (a fact also documented by Griffith et al. (2009)) – potentially because they have less flexibility, in terms of storage, transport or liquidity, to take advantage of sales. This means that their basket of purchases is less exposed to the reductions in the prevalence of promotions that occurred at the beginning of lockdown. The hypothesis that differential usage of promotions is central to explaining lower inflation for the lowest spending quartile is confirmed when we compute inflation for each spending quartile based on only non‐promotion transactions; inflation computed based on non‐promotion transactions is essentially the same across spending quartiles.

## VI. Conclusion

In this paper, we use barcode‐level data covering fast‐moving consumer goods in Great Britain to assess to what extent high‐frequency changes in shopping behaviours may influence and lead to biases in inflation measurement.

We show that the expenditure shares of UPCs changed more than usual during lockdown, but that substitution bias was similar to preceding years. We also show that consumers switched across shopping formats and retailers, but the changes in shopping behaviour and difference in prices across formats and retailers were not large enough to account for a significant portion of the inflationary spike at the start of lockdown. Conversely, changes in promotions were central to driving higher inflation, and the differential use of promotions across the distribution of total spending can account for why lower‐spending households experienced moderately lower inflation than other households.^22^

These findings complement those of Cavallo (2020) and Seiler (2020), who document that changes in expenditure patterns across broad sectors during the pandemic led to an increase in inflation experienced by consumers. Their results are driven by the relative increase in consumption of food and non‐alcoholic beverages, which are more inflationary than other spending categories. While these papers focus on patterns across broad sectors, we study the same channel within the fast‐moving consumer goods sector, using barcode‐level data providing precise measurement of prices and quantities over time.

Overall, our findings indicate that it is crucial for statistical agencies to measure promotions accurately, especially during major economic crises. Within‐sector substitution bias from changes in shopping behaviour appears to be an important but more systematic bias, whose magnitude has remained unchanged during lockdown.

## Supporting information

• Appendix

## Footnotes

1 Given the dramatic recent increase in central banks’ balance sheets, it is particularly important to monitor price stability. Moreover, for the design of transfers and social insurance programmes, it is important to know whether different types of households have experienced different rates of inflation so as to better target those with reduced purchasing power.

2 For example, Cavallo (2020), Diewert and Fox (2020) and Jaravel and O'Connell (2020).

3 Our approach helps alleviate several data limitations from the sectoral‐level studies. First, because goods are more substitutable within than across sectors, analysing the magnitude of substitution bias within sectors (and not just across them) is of particular importance. Second, the changes in sectoral expenditure shares in Cavallo (2020) and Seiler (2020) are inferred from debit card expenditure, which may not appropriately cover certain sectors (for example, car purchases and rental payments are typically not observed). Third, the sectoral‐level price indices used in the Cavallo and Seiler papers may be afflicted by various biases (for example, unobserved changes in quality and within‐sector expenditure switching). In contrast, we directly observe changes in prices and quantities for hundreds of thousands of identical barcodes over time. A comprehensive account of expenditure switching patterns must reflect changes in expenditure both across and within sectors; therefore our within‐sector analysis complements the cross‐sector studies of Cavallo (2020) and Seiler (2020).

4 While inflation and promotion changes are concentrated in particular store types, we find they affect most product categories.

5 National statistical offices’ current practice entails discarding promotions that involve a quantity discount.

6 In prior years, the set of fast‐moving consumer goods covered in our data set accounted for approximately 40 per cent of household expenditures on goods and 15 per cent of total household expenditures on both goods and services (see Jaravel (2019)). The coverage offered by the scanner data should be larger during the Great Lockdown because sectors that are entirely shut down are typically not covered by the scanner data. Cavallo (2020) shows that consumers spent relatively more on food during lockdown.

7 In our analysis, we drop transactions in the top 0.5 per cent of the expenditure or quantity distribution; this does not impact our findings.

8 We do not directly observe store size. Our classification of large and small stores is based on the ‘fascia’ provided in the data. Compact stores include a set of supermarkets with typically small store formats (for example, Co‐op and Somerfield), corner shops and, for the biggest two supermarkets (Sainsbury's and Tesco), their small store formats (identified by the fascia Sainsbury's Local or Tesco Metro).

9 Price indices can be adjusted to account for the impact of product churn on the cost of living, at the expense of placing structural restrictions on the form of the underlying utility function. A prominent choice in the literature is the CES (constant elasticity of substitution) utility function, following Feenstra (1994) and Broda and Weinstein (2010).

10 A downside of chained indices is that they can suffer from chain drift. Intuitively, this problem arises when there is a high‐frequency relationship between changes in price and expenditure weights, which can lead to a chained index either under‐ or over‐stating inflation between two non‐consecutive time periods, relative to a direct comparison between prices and expenditure weights in the two periods. Chained drift is pronounced at the weekly level, but not at the monthly level which we study.

11 Jaravel and O'Connell (2020) consider the implications for inflation of product entry and exit, of using fixed base rather than chained indices, and of alternative superlative indices. Product exit at the onset of lockdown contributes towards an increase in the cost of living. Chained, fixed base and alternative superlative indices all lead to similar conclusions about inflation for continuing products.

12 We compute this as the sum of total expenditure on the product divided by total quantity.

13 In particular, let *i* denote the set of UPCs available over the first five months of the year and *s_i_*_,_ *_t_* the share of total period *t* expenditure on UPC *i*. The dissimilarity index between periods *t* and *t*+1 is defined as $\frac{1}{2}\sum_{i}\left|s_{i,t+1}-s_{i,t}\right|$.

14 It is also instructive to note that the change in the dissimilarity index at the onset of lockdown is larger than that between 18 December to 17 January, which includes the holiday periods, and 18 January to 17 February. This shows that the change in expenditures induced by lockdown is larger than the change observed during the festive holiday period.

15 Our main inflation measure defines products as UPCs. Changes in shopping formats may influence the measured unit price of UPCs available across multiple formats. In Figure A.1 in the online appendix, we compute inflation based on products defined as UPC–store‐format. This strips out the effect of changes in store format from prices in the index. Inflation measured under this alternative product definition is almost identical.

16 In Figure A.1 in the online appendix, we show that when we define products by UPC–retailer (thereby stripping out the influence of retailer switching on unit prices), measured inflation is only modestly below the main measure when products are defined by UPC.

17 For instance, see statement by the UK Competition and Markets Authority – https://www.gov.uk/government/news/covid-19-cma-approach-to-essential-business-cooperation.

18 In Figures A.3 and A.4 of the online appendix, we document heterogeneity in switching across shopping formats and retailer types by spending quartile; higher spending quartiles switched more strongly away from large stores and toward compact stores, and lower spending quartiles switched most strongly away from discounters and toward the big four retailers.

19 See, for example, Kaplan and Schulhofer‐Wohl (2017) and Jaravel (2019).

20 See Hagenaars, de Vos and Zaidi (1994).

21 Note that Jaravel and O'Connell (2020) show a similar pattern holds after controlling for other demographic variables.

22 Multiple factors may have led to the fall in promotions. For example, retailers may need to increase prices because of increased production costs due to interrupted supply chains and to the impact of social distancing restrictions on labour supply, but price hikes during the pandemic may create reputational risks with consumers. Lowering the frequency of promotions may provide a way for retailers to increase effective prices paid without creating as much reputational damage as with more overt price hikes.
