# Supplementary material for: High‐Frequency Changes in Shopping Behaviours, Promotions and the Measurement of Inflation: Evidence from the Great Lockdown
Source: Fisc Stud. 2020 Nov 30;41(3):733–55. doi: 10.1111/1475-5890.12241 (PMC7753338; doi:10.1111/1475-5890.12241)
Supplement: Supplementary file 1 — • Appendix [file FISC-41-733-s001.pdf]

Online Appendix

High-Frequency Changes in Shopping Behaviours, Promotions and the Measurement of Inflation: Evidence from the Great Lockdown

Xavier Jaravel and Martin O'Connell

September, 2020

A Additional tables and figures

Table A.1: *Shopping format shares in 2019, by spending quartile*

	Spending quartile			
	1st	2nd	3rd	4th
Large stores	83.1	80.2	77.3	76.8
Compact stores	7.0	5.5	5.4	5.6
Internet	4.7	10.1	12.7	12.8
Non-food stores	5.2	4.2	4.6	4.8

Note: Numbers show share of expenditure in 2019.

Table A.2: *Retailer type shares in 2019, by spending quartile*

	Expenditure quartile			
	1st	2nd	3rd	4th
Big four	54.9	59.6	63.1	67.4
Discounters	31.9	27.8	23.1	15.8
Premium	2.5	3.9	4.8	7.3
Convenience	5.4	4.3	4.3	4.5
Non-food	5.3	4.4	4.7	5.0

Note: Numbers show share of expenditure in 2019.

Table A.3: *Promotion status, by spending quartile*

	Expenditure quartile			
	1st	2nd	3rd	4th
No promotion	70.5	68.7	67.4	65.8
Price promotion	20.6	21.6	22.4	23.5
Quantity promotion	8.9	9.7	10.2	10.6

Note: Numbers show share of expenditure in 2019.

Figure A.1: *Inflation and shopping behaviours*

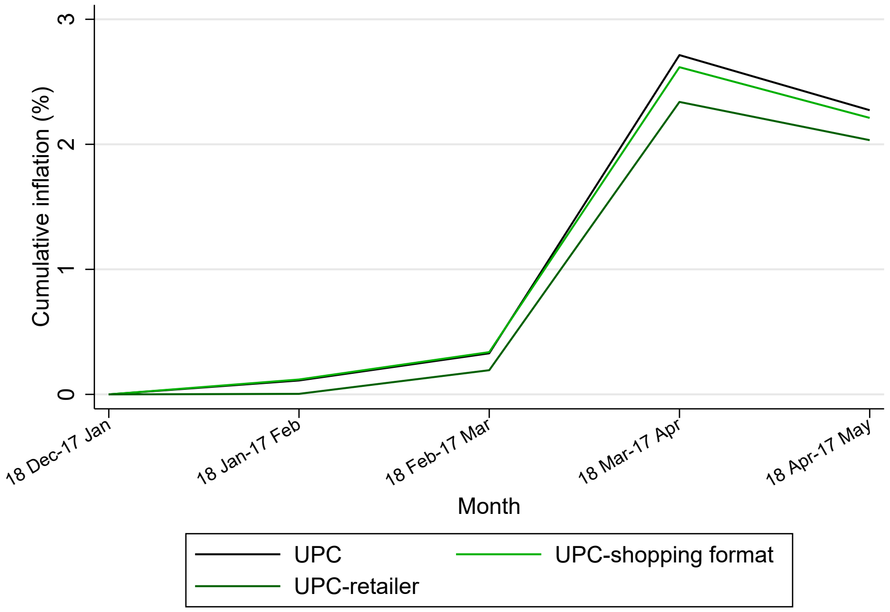

Note: Lines show cumulative monthly inflation measured with a chained Fisher index when products are defined as UPCs, UPC–shopping-format and UPC–retailer. Shopping format is defined in Table 1. Retailers are Aldi, Asda, Co-op, Iceland, Lidl, Marks and Spencer, Morrisons, Ocado, Sainsbury's, Tesco, Waitrose, other food stores and non-food stores.

Figure A.2: *Change in promotions, by product type*

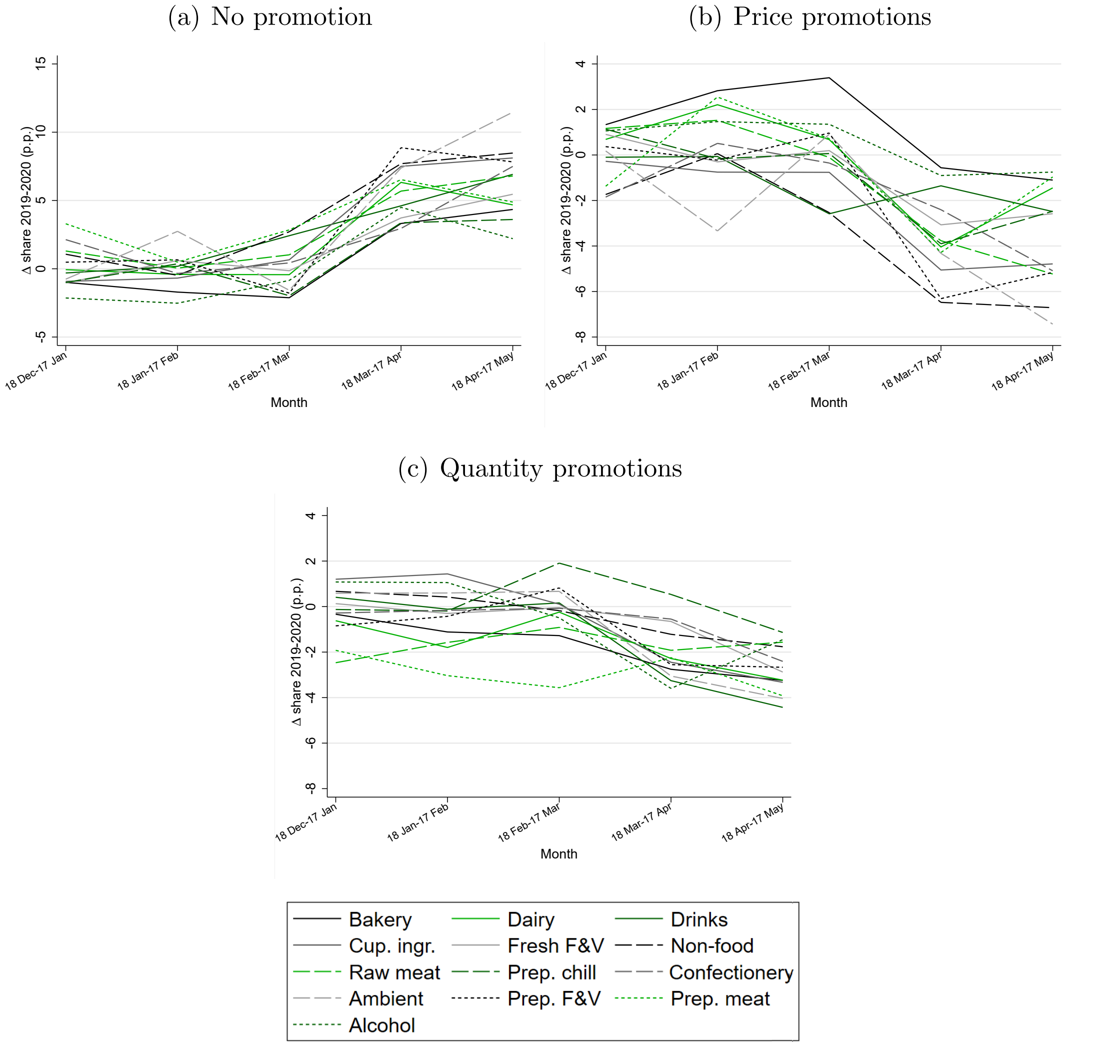

Note: Each panel shows change in expenditure share in percentage points in 2020 relative to 2019. Cup. ingr. stands for cupboard ingredients; F&V stands for fruit and vegetables; Prep. stands for prepared.

Figure A.3: *Change in shopping format, by spending quartile*

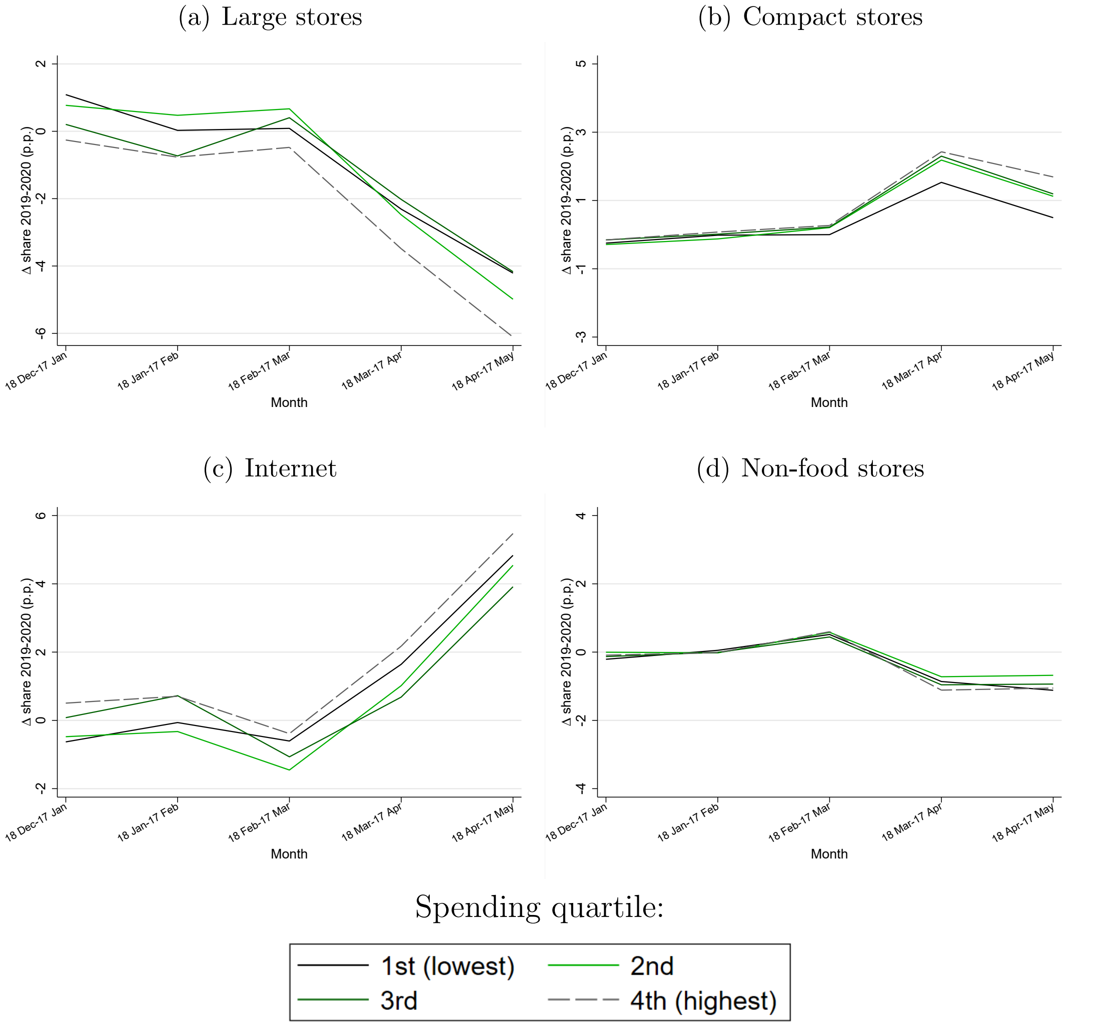

Note: Figure shows change in share of expenditure in large stores, in compact stores, online and in non-food stores in percentage points in 2020 relative to 2019.

Figure A.4: *Change in retailer type, by spending quartile*

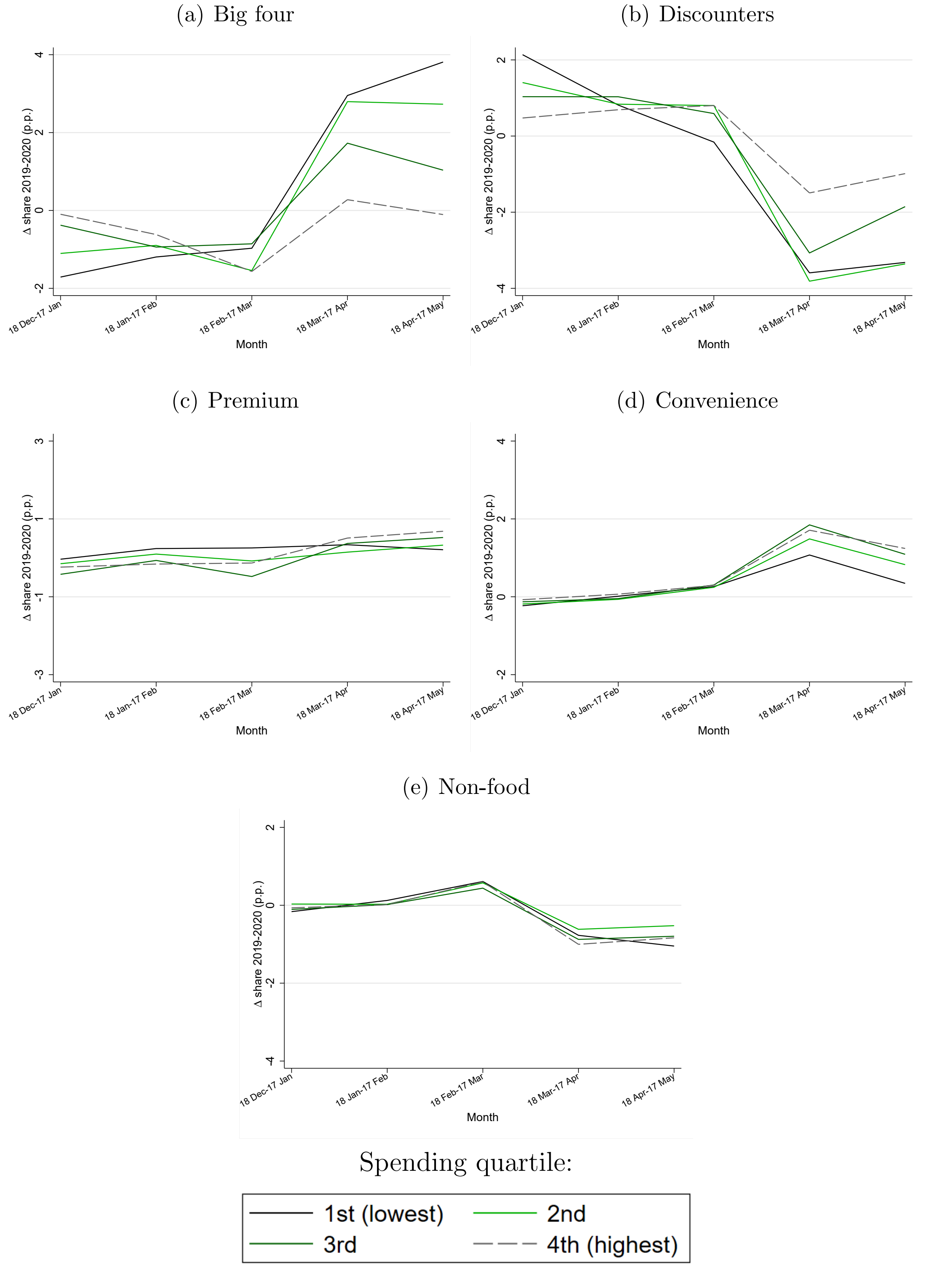

Note: Figure shows change in share of expenditure in the retailer types – big four, discounters, premium, convenience and non-food – in percentage points in 2020 relative to 2019.
